# Thyrotoxic Periodic Paralysis in an Asian Male With Graves' Disease

**DOI:** 10.7759/cureus.96063

**Published:** 2025-11-04

**Authors:** Basimah Mehmood Wardah, Ans Ahmad, Muhammad Masudul Hasan Nuri

**Affiliations:** 1 Nephrology, Tahir Heart Institute, Chenab Nagar, PAK; 2 Cardiology, Tahir Heart Institute, Chenab Nagar, PAK

**Keywords:** anti-acetylcholine receptor antibodies, graves´disease, hyperthyroidism, hypokalemia, hypokalemic paralysis, periodic paralysis, thyrotoxic periodic paralysis, tpp

## Abstract

Thyrotoxic periodic paralysis (TPP) is a rare but potentially life-threatening complication of hyperthyroidism. We present the case of a 27-year-old Asian male with long-standing, untreated hyperthyroidism who presented with recurrent episodes of sudden, reversible muscle weakness. These episodes involved acute flaccid paralysis predominantly affecting the lower limbs and hypokalemia, which improved rapidly with careful potassium replacement. Further investigation confirmed a diffuse toxic goiter consistent with Graves' disease. Interestingly, anti-acetylcholine receptor antibodies were found in the absence of any clinical findings of myasthenia gravis, suggesting a possible autoimmune predisposition. This case underscores the importance of early recognition of TPP to ensure timely management, prevent recurrence, and reduce the risk of complications.

## Introduction

Thyrotoxic periodic paralysis (TPP) is a rare but potentially life-threatening complication of hyperthyroidism, most commonly associated with Graves' disease. It usually presents as sudden, symmetrical paralysis and is characterized by episodes of hypokalemia and muscle weakness. It can progress to quadriplegia and respiratory muscle involvement. The condition mainly involves young males, particularly those of Asian descent, although cases in Western populations are also increasingly recognized [1].

The pathophysiology involves potassium shift from the extracellular to the intracellular space, worsened by the hyperadrenergic state triggered by thyroid hormones. This shift results from the activation of the sodium-potassium pump, whose activity is increased in hyperthyroidism. Factors like high carbohydrate intake, exercise, infections, and corticosteroid use have been identified as triggers for attacks. While the primary cause of hyperthyroidism in most patients is Graves' disease, TPP can also occur in patients with toxic nodular goiter, other forms of thyrotoxicosis, and exogenous thyroxine use. Clinically, TPP may be confused with other causes of sudden paralysis because of its similar symptoms [2].

The characteristic triad of acute hypokalemia, muscle paralysis, and thyrotoxicosis confirms the diagnosis of TPP [3]. Electrocardiogram findings, such as sinus tachycardia, prolonged PR interval, and U-waves, can also aid in diagnosis [4]. Treatment requires immediate potassium repletion; however, caution is necessary to prevent rebound hyperkalemia, which can lead to severe arrhythmias [5]. Long-term management aims to control the underlying hyperthyroidism, primarily through antithyroid treatments like methimazole or propylthiouracil, with other options including thyroidectomy and radioactive iodine therapy [6].

Beta-blockers are effective in the acute phase of TPP because they block adrenergic stimulation of the sodium-potassium pump, helping to restore serum potassium levels without causing rebound hyperkalemia, unlike aggressive potassium supplementation [4]. In conclusion, early recognition and treatment of TPP are essential to avoid life-threatening complications. This condition emphasizes clinicians to maintain a high index of suspicion for thyroid-related causes of acute paralysis, particularly in patients presenting with unexplained hypokalemia and muscle weakness.

## Case presentation

A 27-year-old Asian male with a long-standing history of hyperthyroidism presented with recurrent episodes of lower limb muscle weakness that left him unable to walk. These episodes lasted from a few minutes to an hour. He had normal muscle power between episodes. The ocular and respiratory muscles were unaffected. He also reported persistent palpitations, increased appetite, intermittent diarrhea, and excessive sweating. He had been non-compliant with his prescribed antithyroid medications.

He appeared anxious and exhibited significant muscle wasting. Fine postural tremors were present in both outstretched hands, and his palms were warm and sweaty. Mild exophthalmos and lid lag were also observed. He had tachycardia, with a heart rate of 130 beats per minute, and had systolic hypertension (blood pressure 160/60 mm Hg) with a widened pulse pressure. His visual acuity and extraocular movements were normal.

**Figure 1:**
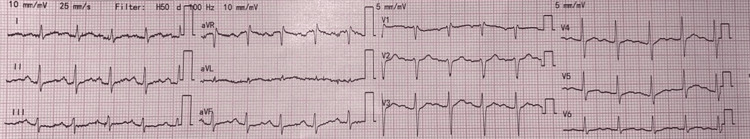
ECG of the patient Electrocardiogram (ECG) showing sinus tachycardia with a heart rate of approximately 130 beats per minute.

During the episode of muscle weakness, his physical exam showed muscle power of 2/5 in both upper limbs and 1/5 in both lower limbs. His investigations showed hypokalemia with a serum potassium level of 2.4 mmol/L. He was carefully given both oral and intravenous potassium replacement, which raised his serum potassium to 3.9 mmol/L. His paralysis fully resolved after his serum potassium levels normalized. The power was 5/5 in all four limbs between these episodes. His ECG showed sinus tachycardia (Figure 1).

Echocardiography was normal with a left ventricular ejection fraction of 70%. Thyroid function tests revealed markedly suppressed thyroid-stimulating hormone (TSH) levels and significantly elevated total T3 and free T4 (Table 1).

**Table 1:** Thyroid profile showing significant hyperthyroidism TSH: thyroid-stimulating hormone, mIU/L: milli-international units per liter, pmol/l: picomoles per liter, nmol/l: nanomoles per liter

Test	Result	Reference value
Serum TSH	< 0.01	0.4 – 4.5 mIU/L
Serum Free T4	42.7	8.0 – 21.0 pmol/l
Serum Total T3	7.9	1.1 – 2.7 nmol/l

**Figure 2:**
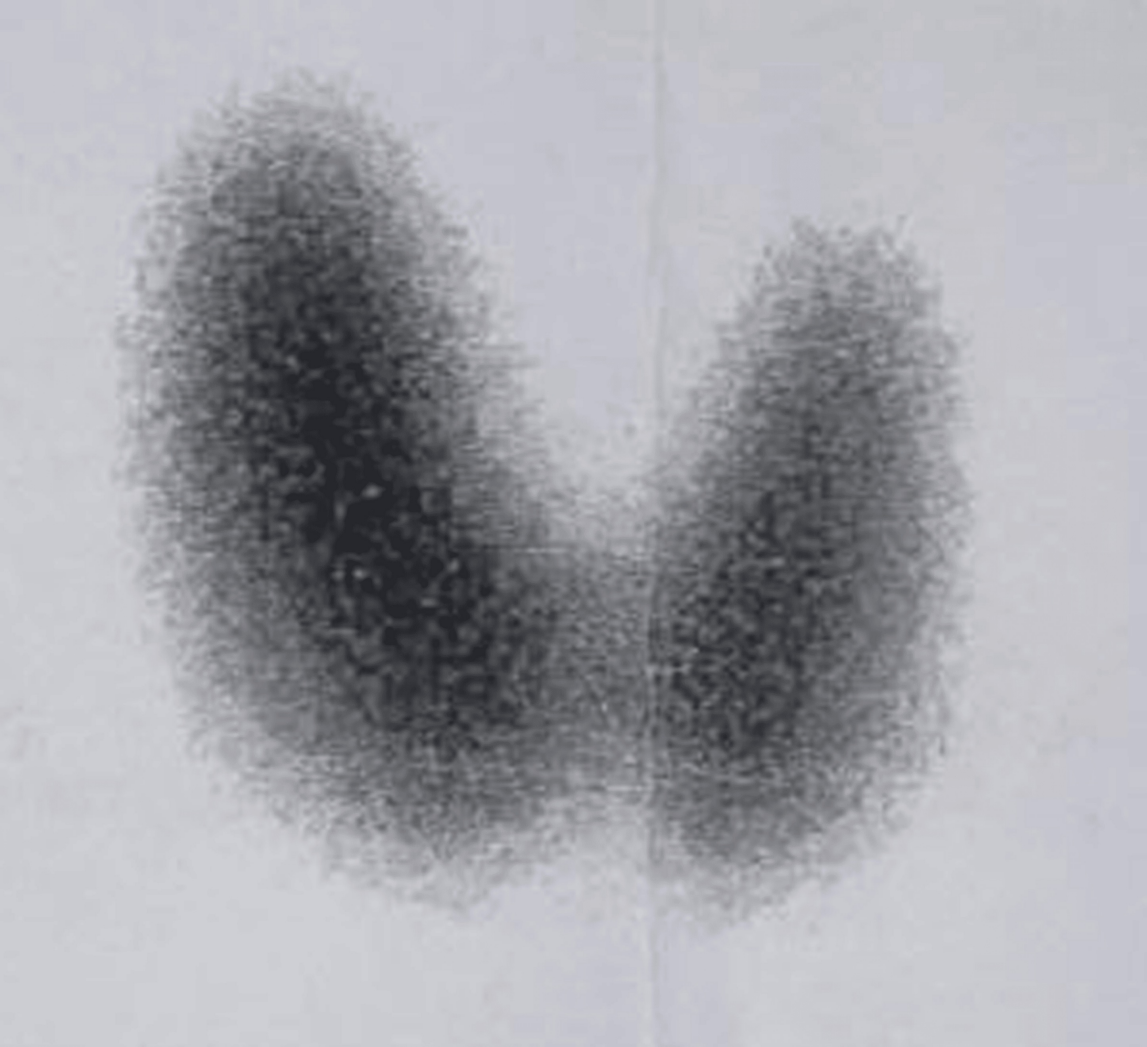
Thyroid scan image Thyroid scan showing diffusely enlarged thyroid gland, indicating diffuse toxic goitre (Graves' disease).

The thyroid scan showed a diffusely enlarged gland with uniform uptake, confirming diffuse toxic goiter (Graves’ disease) (Figure 2).

Notably, anti-acetylcholine receptor antibodies were positive; however, there were no clinical signs of myasthenia gravis (MG). Based on the history, clinical examination, and laboratory findings, the diagnosis of TPP secondary to Graves’ disease was established. His non-compliance with the prescribed treatment led to unchecked thyrotoxicosis associated with frequent episodes of muscle paralysis. He was prescribed carbimazole 20 mg three times daily and propranolol 10 mg three times daily along with counseling regarding medication adherence. He has been scheduled for regular follow-up to monitor for recurrence of paralysis and for any emerging features of MG.

## Discussion

TPP is a rare but serious complication of hyperthyroidism. Unlike most thyroid disorders, which predominantly affect women, it mainly affects men, with the highest prevalence in East Asian populations. The diagnosis of TPP is made when a patient experiences a paralytic attack along with low potassium levels and hyperthyroidism. These episodes of muscle weakness typically occur at rest after heavy exertion or following a high-carbohydrate meal. While TPP isn't directly tied to the severity or duration of hyperthyroidism, it often occurs in people with Graves' Disease. Certain genetic mutations can increase the risk of TPP, with the most significant one affecting the gene that codes for Kir2.6, a potassium channel regulated by thyroid hormone [1].

Thyroid hormones can also increase the sensitivity of beta-adrenergic receptors, which in turn boosts the activity of the sodium-potassium ATPase pump. This facilitates the intracellular shift of potassium, leading to hypokalemia and muscle paralysis. When evaluating a patient with periodic paralysis, it is essential to rule out other conditions that can cause similar symptoms. These include conditions such as familial periodic paralysis, drug-induced paralysis, as well as other causes of sudden muscle weakness like MG and Guillain-Barré Syndrome [7].

MG is an autoimmune disorder affecting the neuromuscular junction, characterized by variable motor weakness. This weakness results from an antibody-driven immune attack targeting proteins in the postsynaptic membrane of the neuromuscular junction [8]. The coexistence of Graves’ disease and MG in the same individual has been observed. Due to their shared pathophysiological pathways, diagnosing and treating these two conditions can be challenging [9]. Our patient also tested positive for acetylcholine receptor antibodies in the absence of clinical findings suggestive of MG. His episodes were therefore attributed to TPP rather than MG. It has been observed that some patients may have positive titres years before MG becomes clinically evident [8]. Although our patient doesn’t have symptomatic MG at present, close follow-up and monitoring are highly needed. 

The management of TPP involves carefully correcting hypokalemia to avoid the potential risk of rebound hyperkalemia, as the total body potassium remains unaffected. Antithyroid drugs and beta blockers are recommended for symptomatic management and to achieve the euthyroid state [10]. Patient education plays a crucial role in preventing recurrent episodes of TPP, emphasizing adherence to prescribed medications and avoidance of precipitating factors such as consuming heavy carbohydrate meals, strenuous exercise, and high sodium intake [11].

## Conclusions

TPP is a rare but potentially life-threatening complication of untreated hyperthyroidism, which is common among young Asian men. Timely diagnosis and treatment are extremely important for resolving paralysis and preventing arrhythmias. Potassium replacement should be carefully planned to avoid rebound hyperkalemia. Nonselective beta-blockers can also be helpful as they decrease adrenergic stimulation that encourages intracellular potassium shift. Long-term management involves antithyroid medications, radioactive iodine, or surgery to achieve a euthyroid state, along with avoidance of precipitating factors. The presence of anti-acetylcholine receptor antibodies in the absence of any clinical signs of MG emphasizes the importance of ongoing monitoring and the potential for coexisting autoimmune conditions.

## References

[1] Isshak R, Orhun N, Rajab I, Shober D, Michael P (2025). Silent paralysis: recurrent thyrotoxic periodic paralysis in a young Hispanic male with Graves’ disease. AACE Endocrinol Diabetes.

[2] Lin SH (2005). Thyrotoxic periodic paralysis. Mayo Clin Proc.

[3] Chang CC, Cheng CJ, Sung CC, Chiueh TS, Lee CH, Chau T, Lin SH (2013). A 10-year analysis of thyrotoxic periodic paralysis in 135 patients: focus on symptomatology and precipitants. Eur J Endocrinol.

[4] Salih M, van Kinschot CM, Peeters RP, de Herder WW, Duschek EJ, van der Linden J, van Noord C (2017). Thyrotoxic periodic paralysis: an unusual presentation of hyperthyroidism. Neth J Med.

[5] Matsui D, Mugikura S, Goshima T, Ishizuka N, Jingushi N, Uenishi N, Iwata M (2025). Rebound hyperkalemia after potassium repletion in thyrotoxic periodic paralysis: a case report and review of management implications. Cureus.

[6] Cid Puente R, Aguirre Moreno PD, Barrios Muñoz AM, Garcia Luis AM, Contreras Saenz CP (2025). Unveiling thyrotoxic periodic paralysis: a rare hyperthyroid complication. Cureus.

[7] Haider M, Chachar AZ, Munir A (2019). Thyrotoxic periodic paralysis. J Ayub Med Coll Abbottabad.

[8] Rousseff RT (2021). Diagnosis of myasthenia gravis. J Clin Med.

[9] Amin S, Aung M, Gandhi FR, Pena Escobar JA, Gulraiz A, Malik BH (2020). Myasthenia gravis and its association with thyroid diseases. Cureus.

[10] Banavathu T, Tripathi S, Sukhadiya P, Ahari K, Meena DS, Garg MK (2021). Thyrotoxic periodic paralysis with thyroid storm as the first presentation of Graves' disease: a case report. Arch Acad Emerg Med.

[11] Siddamreddy S, Dandu VH (2025). Thyrotoxic periodic paralysis. https://www.ncbi.nlm.nih.gov/books/NBK560670/.

